# Respiratory syncytial virus glycoproteins uptake occurs through clathrin-mediated endocytosis in a human epithelial cell line

**DOI:** 10.1186/1743-422X-5-127

**Published:** 2008-10-25

**Authors:** Abel Gutiérrez-Ortega, Carla Sánchez-Hernández, Beatriz Gómez-García

**Affiliations:** 1 Centro de Investigación y Asistencia en Tecnología y Diseño del Estado de Jalisco A.C., Av. Normalistas #800, Colinas de la Normal, C.P. 44270, Guadalajara, Jalisco, México; 2 Centro Universitario de Ciencias Biológicas y Agropecuarias, Universidad de Guadalajara, Carretera Guadalajara-Nogales km 15.5, Las Agujas, C.P. 45110, Zapopan, Jalisco, México; 3 Departamento de Microbiología y Parasitología, Facultad de Medicina, Universidad Nacional Autónoma de México, Circuito Interior, Ciudad Universitaria, DF México, México

## Abstract

Cell-surface viral proteins most frequently enter the cell through clathrin or caveolae endocytosis. Respiratory syncytial virus antigen internalization by immune cells is via caveolin, however, uptake of paramyxovirus cell membrane proteins by non-immune cells is done through clathrin-coated pits. In this work, the uptake of respiratory syncytial virus cell surface glycoproteins by non-immune human epithelial cells was investigated through indirect immunofluorescence with polyclonal anti-RSV antibody and confocal lasser-scanner microscopy. Clathrin and caveolae internalization pathways were monitored through specific inhibitors monodansylcadaverine (MDC) and methyl-beta-cyclodextrin (MBCD), respectively. Internalization of RSV antigens was inhibited by MDC but not by MBCD, implying that clathrin-mediated endocytosis is the major uptake route of RSV antigens by an epithelial human cell line.

## Findings

Respiratory syncytial virus (RSV) is an enveloped, non-segmented negative-stranded RNA virus, classified within the Paramyxoviridae family (genus Pneumovirus). Worldwide, it is implicated in the majority of lower respiratory tract infections in young children and it is a significant pathogen in the elderly and immunocompromised, being airway epithelial cells the main target for viral replication [1]. Its genome encodes two non-structural and nine structural proteins, three of which are transmembrane surface glycoproteins, F, G and SH. The disulfide-bonded protein F (fusion protein) and the large G protein (attachment protein) are the mayor antigenic determinants of the virus and play a crucial role in virus uptake/penetration by the host, while SH inhibits tumor necrosis factor-alpha (TNF-a) signalling [2-5].

Internalization of envelope viral components may be an important posttranslational regulatory mechanism that modulates the surface expression of viral glycoproteins. Spontaneous and anti-viral antibody-mediated endocytosis of cell surface envelope glycoproteins of paramyxovirus in non-immune epithelial cells is clathrin mediated [6]; in contrast, spontaneous endocytosis of RSV antigen in immune dendritic cells is via caveolin [7]. Previously, we reported that RSV antibody-dependent antigen internalization in non-immune epithelial cells is partially inhibited by incubation in hypertonic medium, suggesting the participation of a clathrin-mediated mechanism [8]. With the aim to confirm whether internalization of RSV cell surface antigen-antibody complexes in epithelial cells occurs through clathrin, the present study was undertaken. The uptake of the antigen-antiviral antibody complexes was blocked with specific clathrin and caveolae inhibitors and endocytosis was monitored.

**Figure 1:**
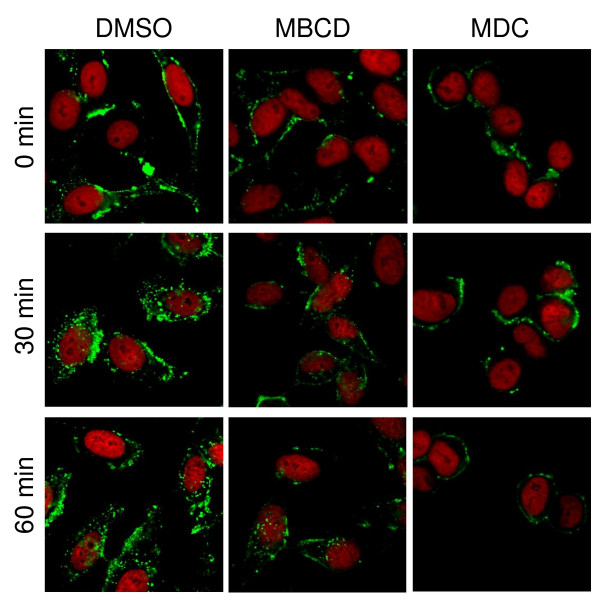
**Endocytosis of RSV envelope proteins in Hep-2 cells**. Hep-2 cells were infected with RSV at a multiplicity of infection of 2. After 12 h, the cells were incubated with anti-RSV goat antibody at 4°C, rinsed and subsequently incubated at 37°C at different times (0, 30 and 60 min) to allow endocytosis to occur. The assay was performed in the presence of diluting agent dimethyl sulfoxide (DMSO), methyl-beta-cyclodextrin (MBCD) or monodansylcadaverine (MDC). The cells were fixed/permeabilized with ice-cold methanol:acetone and incubated with anti-goat IgG-FITC antibody. Later, unbound antibody was washed away and the cells were finally mounted in VectaShield Propidium Iodide medium and analysed on an Olympus FV1000 confocal microscope. RSV antigens and nuclei appear in green and red, respectively.

The endocytosis analysis was performed by confocal lasser-scanner microscopy. For this purpose, HEp-2 cells were RSV infected for 12 h. Origin of cells, virus propagation and infection procedures were previously reported [8]. The infected cells were washed and incubated with medium containing monodansylcadaverine (MDC) or methyl-beta-cyclodextrin (MBCD) at a concentration of 0.2 or 10 mM, respectively, for 30 min at 37°C. Then the cells were washed and incubated in the presence of the inhibitors at the above concentrations with goat polyclonal anti-RSV antibodies (AB 1128; Chemicon) diluted 1:50 in Hanks Balanced Salt Solution (HBSS) for 30 min at 4°C. After the cells were twice washed with ice-cold HBSS, they were immediately incubated at 37°C in pre-warmed medium for 0, 30 and 60 min to allow endocytosis to occur. Then, the cells were fixed and permeabilized with ice-cold methanol-acetone (1:1) for 5 min, blocked with 2.5% bovine serum albumin (Sigma) in phosphate buffered saline (PBS). Internalized primary antibodies were detected with rabbit anti-goat fluorescein-conjugated secondary antibody (81–1620; Zymed) diluted 1:50 in PBS with 1% serum albumin. After extensive washing with PBS, the cells were mounted in Vectashield Propidium Iodide medium (Vector) to visualize counterstained nuclei. As control, infected cells without inhibitors were treated as described. The results are shown in figure 1.

As illustrated in Figure 1, in the absence of endocytosis inhibitors (DMSO treatment), RSV membrane proteins are initially located on the surface of infected cells (0 min), but, as time advances, they are found distributed inside the cells (30 and 60 min). A noteworthy observation is that there was no noticeable difference between 30 and 60 min incubation times. The same happened in RSV-infected cells treated with the caveolae-uptake inhibitor MBCD. On the contrary, when infected cells were treated with the clathrin endocytosis inhibitor, MDC, RSV proteins located to the surface at all incubation times, which indicates that endocytosis of RSV membrane proteins depends on the clathrin-mediated pathway. Clathrin-mediated endocytosis occurs faster in comparison to caveolae-mediated or bulk uptake, which is a consequence of membrane turnover. This explains why RSV proteins did not require more than 30 minutes to be taken up.

Figure 1 also shows that some proportion of RSV protein is still present at the surface of cells, even at the longest incubation time. This observation could indicate that not all RSV envelope glycoproteins are endocytosed. As measured by trypan blue staining, cell viability was unaffected by MDC or MBCD at the concentrations used here (data not shown), suggesting that endocytosis inhibition in MDC-treated cells is not a consequence of pleiotropic effects caused by MDC.

The role of clathrin-mediated uptake in any RSV-related mechanism was not clear until recently. Knockdown of genes associated with clathrin-mediated endocytosis as well as the expression of dominant-negative mutants that inhibit this uptake pathway blocks RSV infection, demonstrating an important role of clathrin for RSV entry [9]. Proteins that are internalized through the clathrin-mediated pathway usually bear the well characterized sorting signal, YXXθ, where X is any amino acid and θ represents any hydrophobic amino acid. This sorting signal was identified in RSV SH glycoprotein as YFTL at its amino terminus located on the cytoplasmic side of the membrane [10], however, there is no current evidence that proves this protein undergoes endocytosis.

In the present work, we confirm that clathrin-mediated endocytosis of RSV envelope proteins bound to antiviral antibodies takes place, but it remains to determine which of the proteins suffers endocytosis. Moreover, we do not know if this phenomenon is linked to an immune evasion mechanism. To this respect, there is a report that correlates internalization of plasma membrane-bound viral glycoproteins proteins to interference with an immune process known as antibody-dependent complement-mediated cell lysis in pseudorabies virus-infected monocytes [11]. The possible biological importance of our finding requires further investigation.

## Competing interests

The authors declare that they have no competing interests.

## Authors' contributions

AGO participated in experimental design, carried out the endocytosis assay and drafted the manuscript. CSH carried out concentration, purification and titration of virus. BGG conceived of the study and helped to draft the manuscript. All authors read and approved the final manuscript.
